# MiR‐19b‐3p accelerates bone loss after spinal cord injury by suppressing osteogenesis via regulating PTEN/Akt/mTOR signalling

**DOI:** 10.1111/jcmm.16159

**Published:** 2020-12-17

**Authors:** Da Liu, Bo Wang, Min Qiu, Ying Huang

**Affiliations:** ^1^ Department of Orthopedics Shengjing Hospital of China Medical University Shenyang People’s Republic of China; ^2^ Department of Ultrasound Shengjing Hospital of China Medical University Shenyang People’s Republic of China

**Keywords:** autophagy, bone loss, miR‐19b‐3p, spinal cord injury

## Abstract

Rapid and extensive bone loss, one of the skeletal complications after spinal cord injury (SCI) occurrence, drastically sacrifices the life quality of SCI patients. It has been demonstrated that microRNA (miRNA) dysfunction plays an important role in the initiation and development of bone loss post‐SCI. Nevertheless, the effect of miR‐19b‐3p on bone loss after SCI is unknown and the accurate mechanism is left to be elucidated. The present work was conducted to explore the role of miR‐19b‐3p/phosphatase and tensin homolog deleted on chromosome ten (PTEN) axis on osteogenesis after SCI and further investigates the underlying mechanisms. We found that miR‐19b‐3p level was increased in the femurs of SCI rats with decreased autophagy. The overexpression of miR‐19b‐3p in bone marrow mesenchymal stem cells (BMSCs) targeted down‐regulation of PTEN expression, facilitated protein kinase B (Akt) and mammalian target of rapamycin (mTOR) phosphorylation, and thereby suppressing BMSCs osteogenic differentiation via autophagy. Besides, the inhibiting effects of miR‐19b‐3p on osteogenic differentiation of BMSCs could be diminished by autophagy inducer rapamycin. Meanwhile, bone loss after SCI in rats was also reversed by antagomir‐19b‐3p treatment, suggesting miR‐19b‐3p was an essential target for osteogenic differentiation via regulating autophagy. These results indicated that miR‐19b‐3p was involved in bone loss after SCI by inhibiting osteogenesis via PTEN/Akt/mTOR signalling pathway.

## 1 INTRODUCTION

Spinal cord injury (SCI) is a terrible neurological disease characterized by sudden sensorimotor dysfunction and autonomic nervous disorder. Severe impactions related to traffic accidents, fall or violence are the primary reasons for SCI and the main manifestations of SCI are very extensive, including paraplegia, quadriplegia, uroschesis and uracratia.^1^, ^2^ As known, the recent clinical investigations have found that rapid bone loss and elevated risk of fracture have close relationships with SCI and appear to be another clinical symptom post‐SCI.^3^, ^4^ The fracture risk of complete SCI patients is 20‐ to 100‐fold higher than the age‐matched ambulatory patients.^5^ In addition, distal femurs and proximal tibias seem to be the most prone to bone loss after SCI.^6^ During the first few months after SCI, rapid bone loss is due to the significant increase in bone resorption accompanied by the deficiency in bone anabolic activity.^7^, ^8^ Therefore, exploring the important factors associated with bone loss after SCI is essential, which may give new insights for alleviating and eventually managing bone loss after SCI.

MicroRNAs (miRNAs) have been reported to be a class of endogenous small non‐coding RNAs with multiple important biological functions. It is well understood that miRNAs specifically bind to the target genes, suppress mRNA translation or accelerate mRNA degradation and thereby down‐regulating their expression.^9^ Growing evidences suggest that miRNA dysfunction is considered as the initiation of various diseases, such as cancer, cardiovascular disorders and osteoporosis.^10^, ^11^, ^12^ Currently, emerging studies indicate that miR‐19b‐3p disorder in the peripheral blood is found in patients with skeletal diseases.^13^, ^14^ The recent study showed that miR‐19b‐3p up‐regulation could be observed in the peripheral blood of patients with non‐traumatic osteonecrosis in the femoral head, the leading cause for the hip joint replacements in young adults.^13^ Inversely, there was a relative low level of miR‐19b‐3p expression in low‐traumatic fracture patients compared with the control individuals.^14^ These events above suggested that miR‐19b‐3p might play an important role in bone metabolic process. Autophagy, a highly conservative physiological process, depends on lysosome‐based recycling pathway referring to cell survival, fate and function, especially during nutrient deprivation.^15^ The protective effects of autophagy on chondrocytes against ageing‐related and experimental osteoarthritis have been identified.^16^, ^17^ Additionally, the disordered autophagy‐lysosome system could be observed in osteoblasts of osteoporosis models and oxidative damage to osteoblasts could be mitigated by early autophagy activation, which might provide a potential therapeutic strategy for osteoporosis.^18^ Accumulating studies indicate that autophagy activation promotes osteogenic differentiation of mesenchymal stem cells via protein kinase B (Akt)/mammalian target of rapamycin (mTOR) cascade, which is an essential part of bone remodelling.^19^ Phosphatase and tensin homolog deleted on chromosome ten (PTEN) has also been reported to implicate in autophagy regulation.^20^ As an inhibitor of Akt, PTEN suppresses Akt activation, inhibits mTOR phosphorylation and thereby disrupting autophagy.^20^ Inspired by the above‐mentioned studies, PTEN might be the pivotal link between miR‐19b‐3p expression and autophagy. Besides, restoring miR‐19b‐3p/PTEN/Akt activation might be a feasible therapeutic strategy for promoting autophagy and alleviating bone loss related to SCI.

For this purpose, we observed miR‐19b‐3p expression in SCI rats and further studied the influence of miR‐19b‐3p/PTEN/autophagy system on osteogenic differentiation of mesenchymal stem cells.

## 2 MATERIALS AND METHODS

### 2.1 Establishment of SCI model

A total of 24 adult male Sprague Dawley (SD) rats weighting 200‐250 g were provided by Liaoning Changsheng biotechnology Co., Ltd. (China). The rats were kept in standard laboratory environment (12 hours light‐dark cycle; temperature 22 ± 1°C; humidity 45%‐55%) with food and water accessible before exposure to any experiment. After the adaptation, the rats were randomly divided into the following two groups:

- SCI group (12 rats exposed to SCI surgery (6 rats euthanized at day 1 after SCI surgery and 6 rats euthanized at day 21 after SCI surgery));
- Control (12 rats exposed to T8 laminectomy only (6 rats euthanized at day 1 after T8 laminectomy and 6 rats euthanized at day 21 after T8 laminectomy)).

The procedures of SCI surgery were in consistent with the previous study.^21^ Briefly, the T8 vertebra was positioned and the T8 laminectomy was orderly implemented after the rat was anaesthetized. Afterwards, the injury of spinal cord was induced by a self‐made impactor (Weight: 30 g; terminal diameter: 3 mm) vertically dropped from a height of 5 cm. After the surgery, all the post‐operative care was in according with the preceding description.^22^ Rats subjected to T8 laminectomy without impaction served as the control. The impairments of motor function in SCI rats were monitored on 0, 1, 4, 7, 14 and 21 day post‐SCI surgery according to the Basso‐Beattie‐Bresnahan (BBB) locomotion scale.^23^ The rats were, respectively, euthanized at day 1 and day 21 post‐SCI surgery and bilateral femurs were collected for the future experiments. Bone mineral density (BMD) of the femur was monitored at day 1 and day 21 post‐injury. All the experimental procedures abided by the Guide for Care and Use of Laboratory Animals and approved by Shengjing Hospital of China Medical University.

### 2.2 Bone marrow mesenchymal stem cells (BMSCs) isolation and treatment

Rat BMSCs were collected from the femurs and tibias of healthy SD rats as previously published with appropriate modifications.^24^ The separated BMSCs were cultured in fresh DMEM media (Gibco, USA) containing 10% foetal bovine serum (FBS, Hyclone, USA) until passage three. BMSCs were transduced with miR‐19b‐3p expressing lentivirus or control lentivirus according to the manufacturer's introduction. The transduced BMSCs were grown in osteogenic differentiation medium (DMEM media (Gibco, USA) containing 10% FBS (Hyclone, USA), 50 μg/mL ascorbic acid, 10 mmol/L sodium β‐glycerophosphate and 10 nmol/L dexamethasone) for 21 days to differentiate into osteoblasts. The differentiated BMSCs were used in the future detection.

BMSCs were incubated in 100 nmol\L rapamycin to active autophagy 24 hours after transduction; afterwards, BMSCs were grown in osteogenic differentiation medium for 21 days to differentiate into osteoblasts. The differentiated BMSCs were used in the future detection.

### 2.3 Dual‐luciferase reporter assay

The possible targeting relationship between miR‐19b‐3p and PTEN mRNA was predicted by using TargetScan (www.targetscan.org). HEK293T cells were seeded in a 12‐well plate and co‐transfected with miR‐19b‐3p mimics or control mimics and PTEN 3'‐UTR wild‐type (wt) or PTEN 3'‐UTR mutant‐type (mut) luciferase reporter plasmid (Wanlei Biological Technology Co., Ltd., China) when the confluence reached 70%. Afterwards, luciferase assays were conducted by using the Dual‐Luciferase assay kit (KeyGEN BioTECH, China) and normalized by Renilla luciferase assay.

### 2.4 Antagomir‐19b‐3p treatment in SCI rats

A total of 12 adult male SCI rats were randomly divided into the following two groups:

- Antagomir‐19b‐3p group (six rats exposed to SCI surgery and antagomir‐19b‐3p treatment);
- Control antagomir group (six rats exposed to SCI surgery and control antagomir treatment).

Following the SCI surgery, the rats received intrathecal injection of antagomir‐19b‐3p (0.5 nmol\L, 5 μL) or control antagomir (0.5 nmol\L, 5 μL) by a PE‐10 catheter (inner diameter: 0.28 mm, external diameter: 0.64 mm) for three days. BMD of the femur was monitored at day 21 post‐injury and the femurs were collected for subsequent experiments. The sequences for antagomir (GenePharma Co., Ltd, Shanghai, China) were as follows:

antagomir‐19b‐3p: 5’‐UCAGUUUUGCAUGGAUUUGCACA‐3’;

control antagomir: 5’‐CAGUACUUUUGUGUAGUACAA‐3’.

### 2.5 BMD detection

The femurs collected at indicated time were carefully cleaned, washed with saline and wrapped in gauze infiltrated with saline. Afterwards, dual‐energy X‐ray absorptiometry (DEXA) was used to detect BMD changes in the femurs. The value of BMD was represented as g/cm^2^.

### 2.6 Histological analysis

The obtained femurs were fixed in 4% paraformaldehyde solution, demineralized, embedded in paraffin and sectioned by rotary microtome into slices at 5 μm thickness. The femur sections were subjected to haematoxylin solution (Solarbio, China) and counterstained with eosin (Sangon, China) as the description of the manufacturer's instruction. Histological changes in the femurs were observed under a light microscopy at 200 × magnification.

### 2.7 Immunohistochemistry

The aforementioned femur sections were subjected to immunohistochemical procedures, which were all based on the published experiment with appropriate modifications.^25^ In brief, the femur sections were orderly incubated in 3% H_2_O_2_ for 15 minutes, goat serum for 15 minutes at room and the primary antibodies (Rabbit anti‐osterix, dilution: 1:200, Abcam, UK; Rabbit anti‐osteocalcin, dilution: 1:200, ABclonal, China) overnight at 4°C. After wash with phosphate buffered solution, the samples were treated with the secondary antibody (dilution: 1:500, Thermo Fisher, USA) for additional 30 minutes at room temperature, diaminobenzidine slides (Solarbio, China) and haematoxylin (Solarbio, China). Finally, the expressions of target proteins were observed under a light microscopy at 400 × magnification.

### 2.8 Immunofluorescence

Immunofluorescence staining targeting LC3 expression in the femur was implemented. The above‐mentioned femur sections were blocked with goat serum for 15 minutes at room, incubated in primary antibody (mouse anti‐LC3, dilution: 1:50, Santa, USA) overnight at 4°C and then FITC‐conjugated secondary antibody (dilution: 1:200, Beyotime Institute of Biotechnology, China) for 1 hour at room temperature. Subsequently, 4', 6‐diamidino‐2‐phenylindole (DAPI, Beyotime Institute of Biotechnology, China) was utilized to label the nuclei. Ultimately, pictures were shot under a fluorescence microscope at 400 × magnification.

### 2.9 RT‐PCR

The total RNA was extracted from femur tissues at the indicated time or differentiated BMSCs using TRIpure (TianGEN, China) and transcribed into the relevant cDNA by the Super MMLV Reverse Transcriptase (TianGEN, China). Stem‐loop RT‐PCR was used to quantify miR‐19b‐3p. RT‐PCR conducted in Exicycler 96 System (Bioneer, Korea) using 2 × Power Taq PCR MasterMix (TianGEN, China) and SYBR Green (Solarbio, China). The levels of miR‐19b‐3p were presented as relative expression, which calculated by comparing with the control group. Primers were as follows:

rno‐miR‐19b‐3p forward: 5'‐TGTGCAAATCCATGCAAAACTGA‐3';

reverse: 5'‐GCAGGGTCCGAGGTATTC‐3';

#### 2.9.1 Western blot

The total proteins of femur tissues or BMSCs were separately extracted, quantified by the BCA kit (Solarbio, China) and separated by sodium dodecyl sulphate polyacrylamide gel (SDS‐PAGE). The separated proteins were electrophoretically transferred onto a polyvinylidene difluoride (PVDF) membrane. After blockage in 5% skimmed milk for 1 hour at the room temperature, the membranes were orderly incubated with homologous primary antibodies overnight at 4°C and diluted horseradish peroxidase (HRP) labelled secondary antibody (dilution: 1:3000, Solarbio, China) for 45 minutes at 37°C. Hereafter, chemiluminescence (ECL) kits (Solarbio, China) were used to visualize the target proteins. Finally, the integrated intensity of the band was quantified by Gel‐Pro‐Analyzer. The protein levels were presented as relative expression, which calculated by comparing with the control group. The primary antibodies were as follows: Rabbit anti‐osterix (dilution: 1:2000); osteocalcin (dilution: 1:1000); PTEN (dilution: 1:1000); p‐Akt (dilution: 1:1000); Akt (dilution: 1:1000); p‐mTOR (dilution: 1:1000); mTOR (dilution: 1:1000); LC3II/I (dilution: 1:1000, Affinity, China); P62 (dilution: 1:2000); Beclin‐1 (dilution: 1:5000); and mouse anti‐GAPDH (dilution: 1:10 000, Proteintech, China).

#### 2.9.2 Statistical analysis

Data were represented as means ± standard derivations (SD) and analysed by Student's *t* test (for two groups). *P* < .05 was considered statistically significant. All the in vivo experiments were independently repeated for six times. All the in vitro experiments were biologically repeated for three times.

## 3 RESULTS

### 3.1 MiR‐19b‐3p expression and autophagy change in SCI rats

**FIGURE 1:**
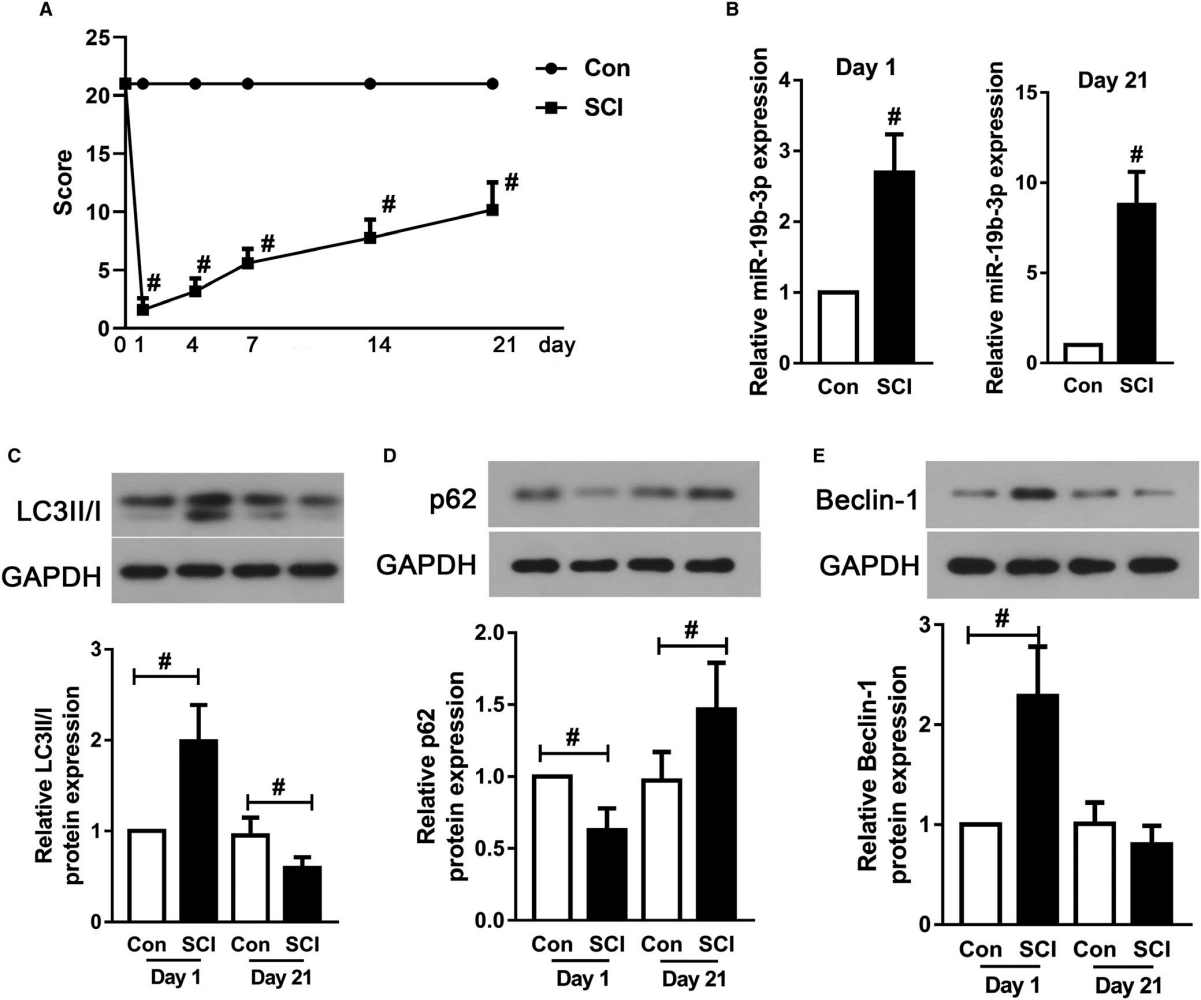
MiR‐19b‐3p expression and autophagy change in SCI rats. (A) The average Basso‐Beattie‐Bresnahan (BBB) scores at 0‐, 1‐, 4‐, 7‐, 14‐ and 21‐day post‐SCI. (B) RT‐PCR was used to detect miR‐19b‐3p expression in the femurs at day 1 and day 21 post‐SCI surgery. Western blot for (C) LC3II/I, (D) p62 and (E) Beclin‐1 in the femurs at day 1 and day 21 post‐SCI surgery. Data were presented as means ± SD, and the statistical difference between two groups was determined by Student's *t* test. ^#^ *P* < .05 vs. Con. All the in vivo experiments were independently repeated for six times (n = 6)

Firstly, SCI models were established in rats and the motor function was detected. As described in Figure 1A, motor function impairments reflected by the decreased BBB scores were elicited by SCI surgery (*P* < .05). As shown in Figure 1B, miR‐19b‐3p expression was significantly increased since the first day after SCI surgery (*P* < .05), besides, the elevated miR‐19b‐3p expression could be maintained until day 21 after SCI surgery (*P* < .05). On the first day after SCI, P62 accumulation was decreased, while beclin‐1 expression and LC‐3II degradation were increased in the femurs, which might be the reason for the regulation of bodies under stress (Figure 1C‐E, *P* < .05). Interestingly, this phenomenon was reversed at day 21 post‐SCI. The reason for the contrary tendency of autophagy at day 1 and 21 post‐injury might be the stress response of the body to SCI stimulation. The data indicated that abnormal expression of miR‐19b‐3p might participate in bone metabolism after SCI by regulating autophagy and subsequent experiments were needed to detect the function of miR‐19b‐3p.

### 3.2 MiR‐19b‐3p overexpression inhibited osteogenic differentiation of BMSCs

**FIGURE 2:**
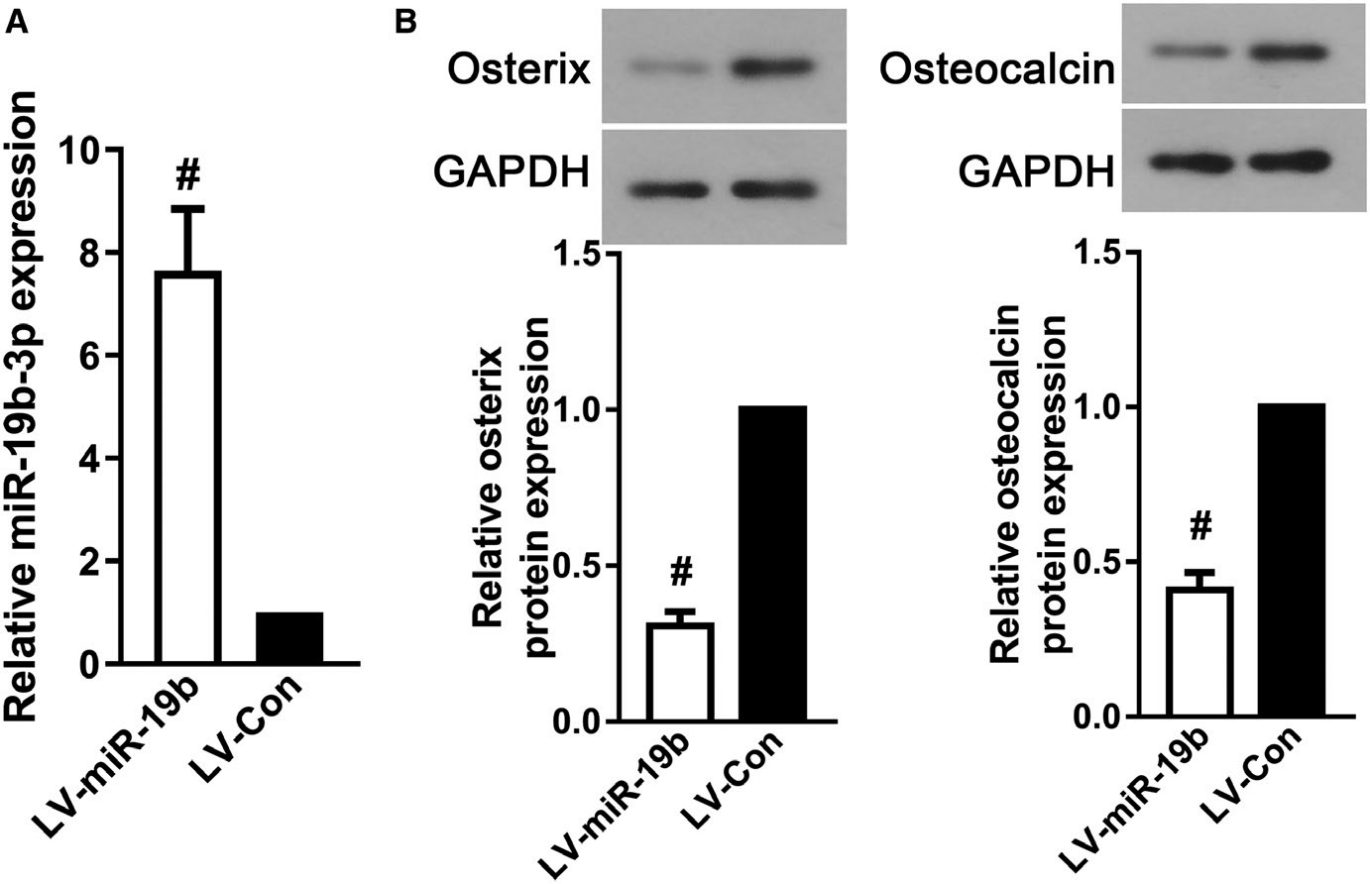
MiR‐19b‐3p overexpression inhibited osteogenic differentiation of BMSCs. (A) RT‐PCR was used to detect miR‐19b‐3p expression in BMSC‐derived osteoblast after transduction. Western blot for (B) osterix andosteocalcin in BMSC‐derived osteoblasts after transduction. Data were presented as means ± SD, and the statistical difference between two groups was determined by Student's *t* test. ^#^ *P* < .05 vs. LV‐Con. All the in vitro experiments were biologically repeated for three times (n = 3)

Since maintaining the function of osteoblasts is necessary to maintain normal bone metabolism, we examined the effects of miR‐19b‐3p expression on osteogenic differentiation of BMSCs. We firstly transduced miR‐19b‐3p expressing lentivirus into BMSCs and the data showed that miR‐19b‐3p expression was observably increased in BMSC‐derived osteoblasts (Figure 2A, *P* < .05). As described in Figure 2B, the expressions of osterix and osteocalcin, markers of osteogenic differentiation, were refrained with miR‐19b‐3p overexpression (*P* < .05). The data indicated that miR‐19b‐3p overexpression suppressed osteogenic differentiation of BMSCs.

### 3.3 MiR‐19b‐3p overexpression suppressed autophagy via binging to PTEN

**FIGURE 3:**
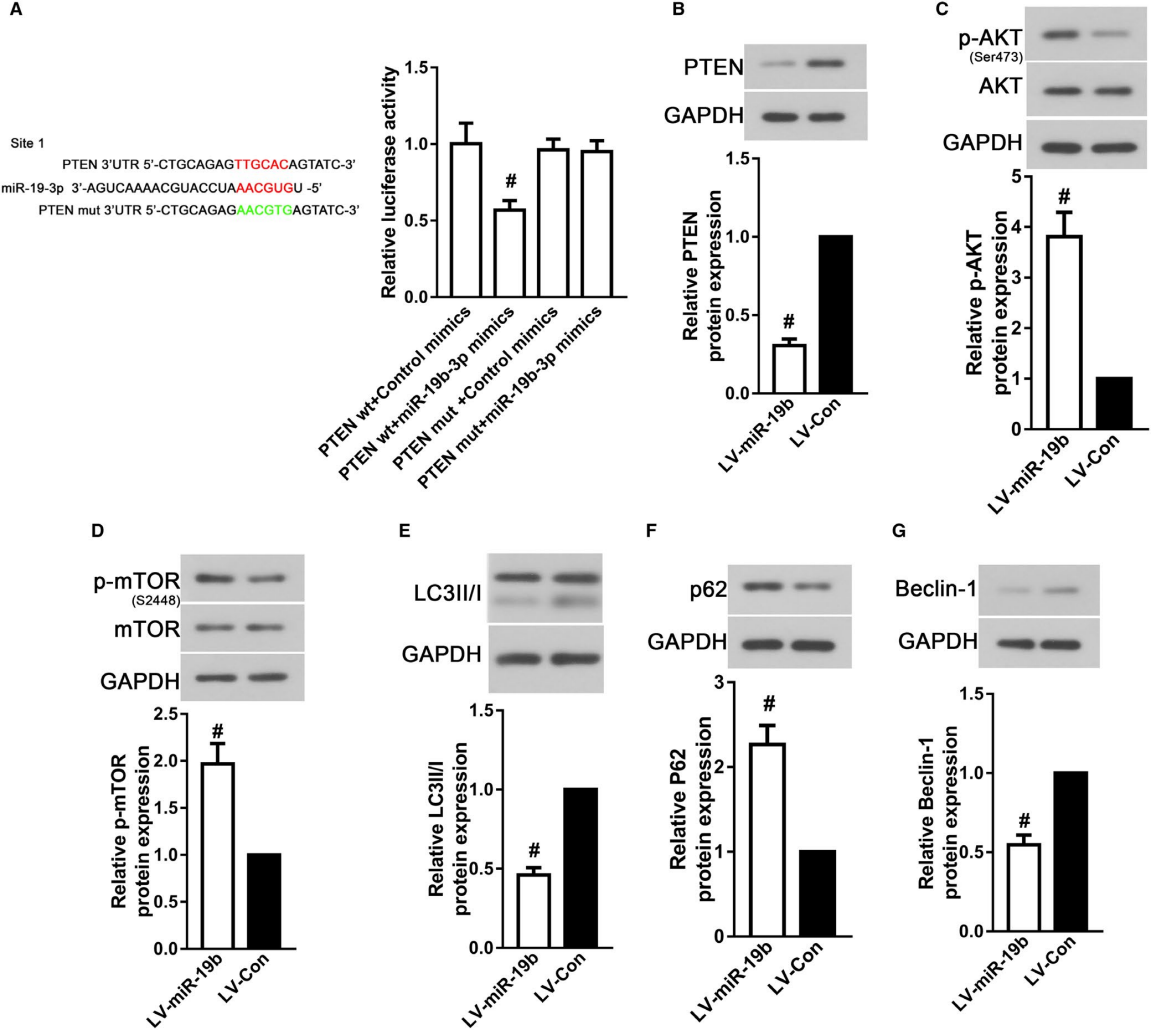
MiR‐19b‐3p overexpression suppressed autophagy via binging to PTEN. (A) Dual‐Luciferase reporter assay was conducted to verify the targeted relationship between miR‐19b‐3p and 3’‐UTR of PTEN in HEK293T cells. Western blot for (B) PTEN, (C) p‐Akt, (D) p‐mTOR, (E) LC3II/I, (F) p62 and (G) Beclin‐1 in BMSC‐derived osteoblasts after transduction. Data were presented as means ± SD, and the statistical difference between two groups was determined by Student's t test. ^#^ *P* < .05 vs. LV‐Con. All the in vitro experiments were biologically repeated for three times (n = 3)

As predicted by TargetScan (www.targetscan.org), PTEN is one of the potential targets of miR‐19b‐3p. We firstly verified the targeting relationship between PTEN and miR‐19b‐3p. As shown in Figure 3A and B, the result of dual‐luciferase reporter assay exhibited that miR‐19b‐3p targeted PTEN expression via binding to its 3'‐UTR (*P* < .05). Since autophagy is a key point in the process of osteogenic differentiation of BMSCs, the effects of miR‐19b‐3p expression on autophagy in BMSC‐derived osteoblasts were testified. Moreover, miR‐19b‐3p overexpression greatly increased LC3II degradation and P62 accumulation, while decreased Beclin‐1 expression in BMSC‐derived osteoblasts compared with the control (Figure 3E‐G, *P* < .05), suggesting miR‐19b‐3p inhibited osteogenic differentiation of BMSCs through regulating autophagy. Autophagy inhibition induced by miR‐19b‐3p overexpression was accompanied by decreased phosphorylation of AKT and mTOR (Figure 3C and D, *P* < .05). The data indicated that miR‐19b‐3p overexpression suppressed autophagy via targeting PTEN.

### 3.4 Activating autophagy abrogated the inhibiting effect of miR‐19b‐3p overexpression on osteogenic differentiation

**FIGURE 4:**
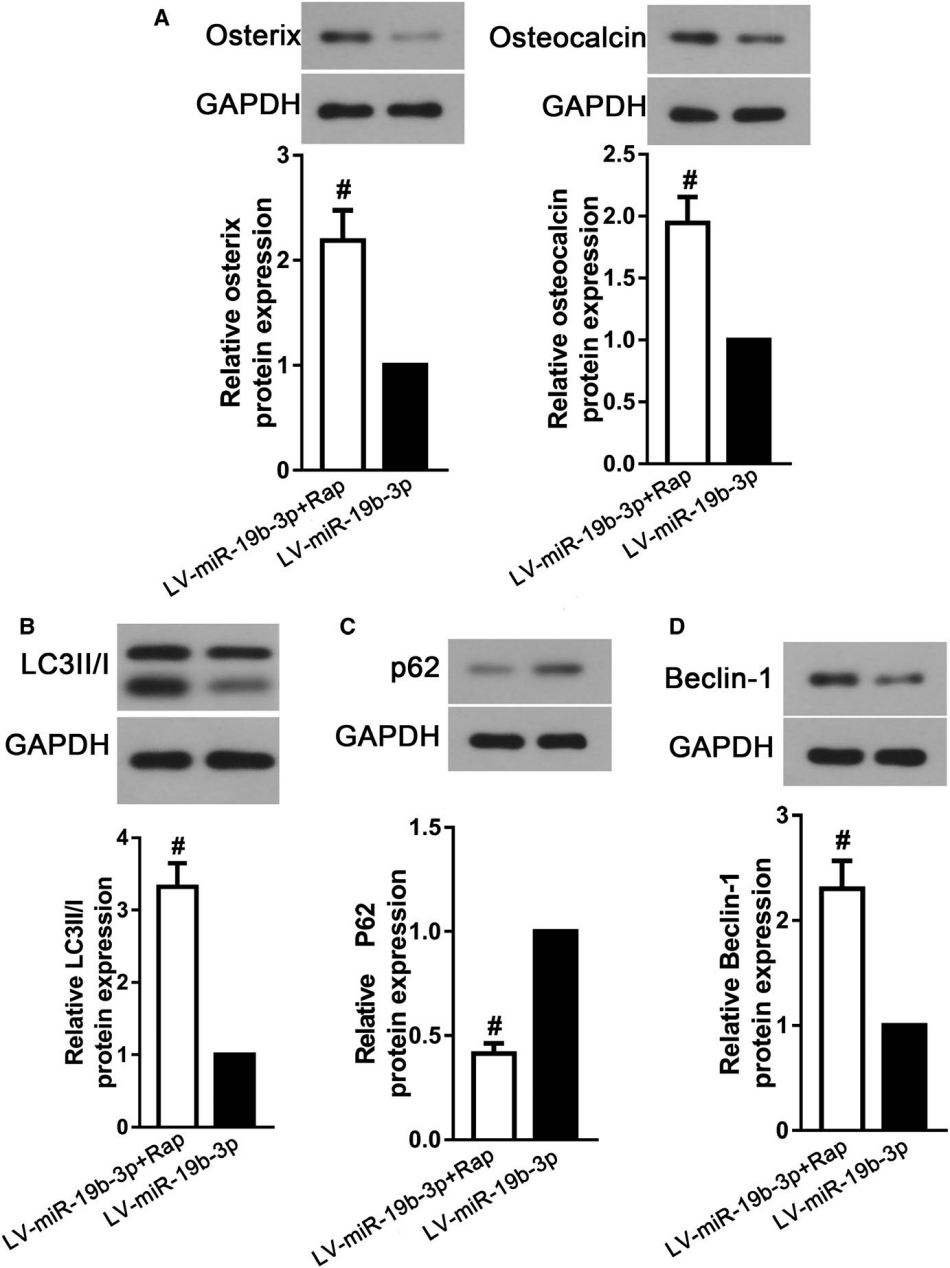
Activating autophagy abrogated the inhibiting effect of miR‐19b‐3p overexpression on osteogenic differentiation. Western blot for (A) osterix, osteocalcin, (B) LC3II/I, (C) p62 and (D) Beclin‐1 in BMSC‐derived osteoblasts after rapamycin treatment. Data were presented as means ± SD, and the statistical difference between two groups was determined by Student's *t* test. ^#^ *P* < .05 vs. LV‐miR‐19b‐3p. All the in vitro experiments were biologically repeated for three times (n = 3)

To verify whether miR‐19b‐3p over‐expression participated in osteogenic differentiation of BMSCs via regulating autophagy, we treated BMSCs with autophagy activator rapamycin after miR‐19b‐3p expressing lentivirus transduction. As described in Figure 4A, compared with the miR‐19b‐3p expressing lentivirus‐treated group, the expressions of osterix and osteocalcin, osteogenic differentiation markers, were dramatically increased after rapamycin incubation (*P* < .05). Moreover, autophagy activator rapamycin reversed the declined autophagy induced by miR‐19b‐3p over‐expression in BMSC‐derived osteoblasts (Figure 4B‐D, *P* < .05). These results indicated that autophagy activation impeded the down‐regulation of osteogenic differentiation induced by miR‐19b‐3p over‐expression.

### 3.5 MiR‐19b‐3p inhibition alleviated SCI‐induced bone loss in rats

**FIGURE 5:**
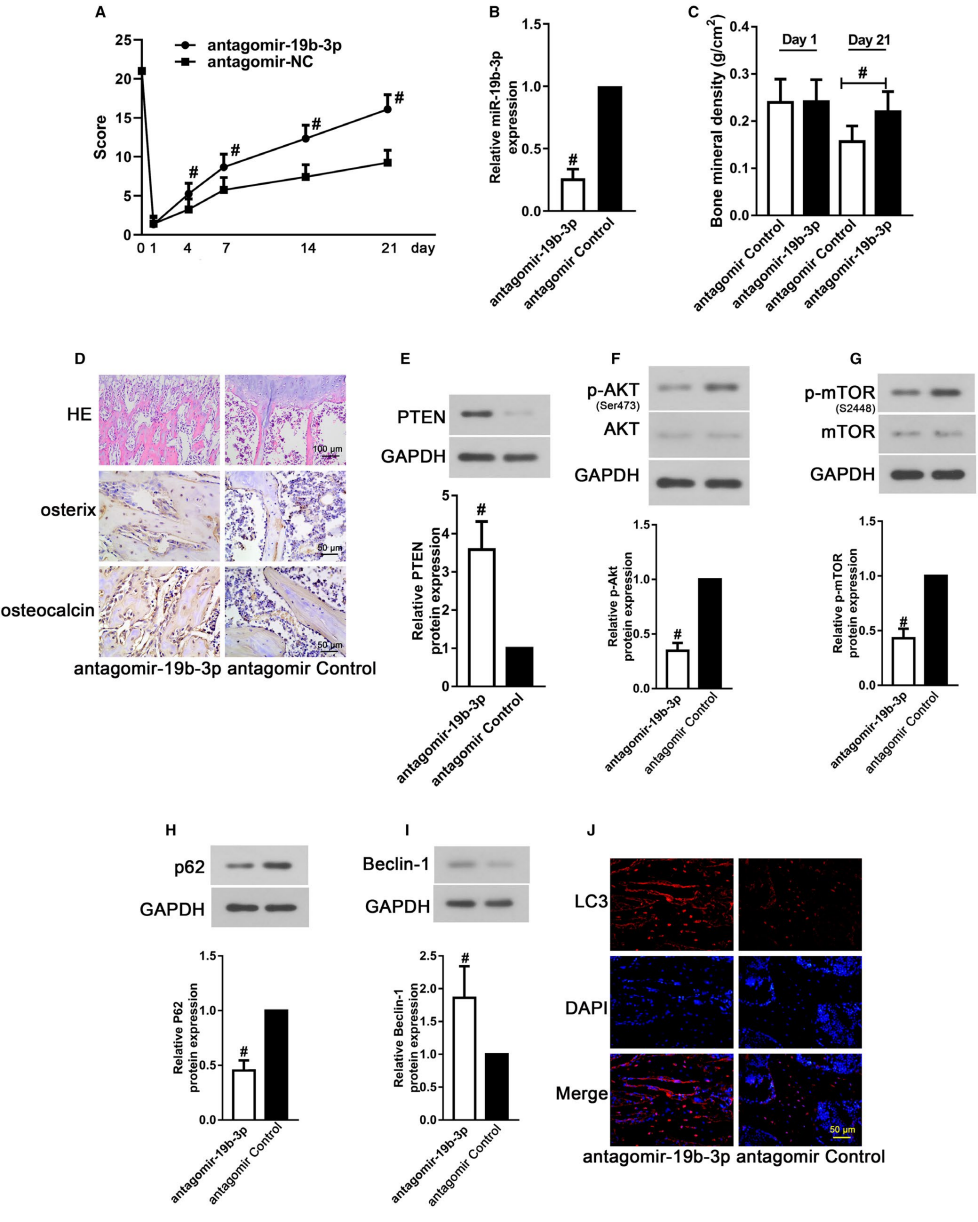
MiR‐19b‐3p inhibition alleviated SCI‐induced bone loss in rats. (A) The average BBB scores at 0, 1, 4, 7, 14 and 21 day post‐SCI. (B) RT‐PCR was used to detect miR‐19b‐3p expression in the femur tissues of SCI rats after antagomir‐19b‐3p treatment. (C) Bone mineral density of SCI rats with antagomir‐19b‐3p treatment. (D) H&E staining (at 200 X magnification) and immunohistochemical staining (at 400 X magnification) targeting osterix and osteocalcin in the femur tissues of SCI rats after antagomir‐19b‐3p treatment. Western blot for (E) PTEN, (F) p‐Akt, (G) p‐mTOR, (H) p62 and (I) Beclin‐1 in the femur tissues of SCI rats after antagomir‐19b‐3p treatment. (J) Immunofluorescence staining for LC3 in the femur tissues of SCI rats after antagomir‐19b‐3p treatment (at 400 × magnification). Data were presented as means ± SD, and the statistical difference between two groups was determined by Student's *t* test. ^#^ *P* < .05 vs. control antagomir group. All the in vivo experiments were independently repeated for six times (n = 6)

The effects of miR‐19b‐3p inhibition on locomotor function and bone loss were certified in SCI rats. As exhibited in Figure 5A and B, the locomotor function of antagomir‐19b‐3p‐treated SCI rats was improved with accompanied by decreased miR‐19b‐3p expression in the femurs (*P* < .05). Besides, the bone mineral density of SCI rats increased at 21 days after SCI surgery compared with the control antagomir‐treated SCI rats (Figure 5C, *P* < .05). In addition, the expressions of osteogenic differentiation markers, including osterix and osteocalcin, were memorably elevated after inhibiting miR‐19b‐3p accompanied by reduced osteocyte lacunae (Figure 5D, *P* < .05). To prove the importance of PTEN and its downstream regulators in the pathogenesis, we evaluated the expressions of PTEN and the phosphorylation of Akt and mTOR in the femur tissues. As shown in Figure 5E‐G, antagomir‐19b‐3p treatment increased PTEN expression, while decreased Akt and mTOR phosphorylation in the femurs after SCI surgery at 21 days post‐injury (*P* < .05). Besides, the levels of P62, Beclin‐1 and LC3 were also detected and the results were shown in Figure 5H‐J. The changes in P62, Beclin‐1 and LC3 expression were also reversed by miR‐19b‐3p inhibition (*P* < .05). The results manifested that miR‐19b‐3p inhibition alleviated SCI‐related bone loss through regulating autophagy via targeting PTEN expression.

## 4 DISCUSSION

In the present study, we demonstrated the facilitating effects of miR‐19b‐3p on bone loss after SCI and investigated the underlying mechanism. Our data showed that miR‐19b‐3p levels were elevated in the femurs of SCI rats, which was accompanied by autophagy inhibition in BMSC‐derived osteoblasts. MiR‐19b‐3p over‐expression restrained PTEN expression, promoted Akt and mTOR phosphorylation, disturbed autophagy flux, and thereby suppressing osteogenic differentiation of BMSCs. Additionally, bone loss after SCI in rats could be reversed by miR‐19b‐3p inhibition. Hence, our data manifested that miR‐19b‐3p could be an attractive target in the prevention of SCI‐related bone loss through regulating autophagy via PTEN/Akt/mTOR signalling pathway.

The SCI rat model was established by using the method of weight‐drop contusion on spinal cord, which was widely used in evaluating the therapeutic effect for SCI and its complications.^26^ In our study, the expressions of miR‐19b‐3p in the femur tissues were monitored at day 1 and day 21 post‐SCI surgery and we found that SCI surgery sharply increased femur miR‐19b‐3p levels. It was interesting to note that enhanced autophagy was found at day 1 after SCI surgery, which was represented by decreased P62 accumulation and increased LC3II/I and Beclin‐1 expression. However, autophagy decreased significantly at day 21, possibly because autophagy was a self‐protective procedure that the body might initiate at the beginning of SCI.^27^ Notably, our results indicated that miR‐19b‐3p might functioned as a promoting factor for SCI‐related bone loss via regulating autophagy. However, the accurate effects and potential mechanisms that miR‐19b‐3p involves in the development of bone loss post‐SCI are still left unclear.

It is generally accepted that bone remodelling, a dynamic physiological process, mainly involves two parts, including bone formation by osteoblasts and bone matrix resorption by osteoclasts.^28^, ^29^, ^30^, ^31^ Osteoblasts are terminally differentiated cells with a short lifespan of approximately three months; hence, new osteoblasts differentiated from endogenous mesenchymal stem cells continue to replace the old ones to maintain the balance of bone growth and bone resorption.^32^, ^33^ The in vivo experiment found miR‐19b‐3p levels were improved after SCI, in order to investigate the possible mechanism whether miR‐19b‐3p was involved in osteogenic differentiation, miR‐19b‐3p expressing lentivirus was transduced into BMSCs before exposure to osteogenic differentiation medium. Fortunately, the osteogenic differentiation ability of BMSCs was decreased with miR‐19b‐3p over‐expression, which was in accordance with the phenomenon of in vivo experiments. The above results indicated that miR‐19b‐3p inhibited osteogenic differentiation of BMSCs.

Since plenty of intracellular pathways at molecular levels influence SCI‐related osteogenesis decrease, we simply studied the signalling associated with miR‐19b‐3p expression. PTEN, an identified tumour suppressor, plays a vital role in mature organisms.^34^ In addition, the distinct roles of PTEN in proliferation and differentiation of multiple cell types have been accepted.^35^ It has been observed that PTEN deletion not only inhibits neural stem cells death, but also facilitates neural stem cells differentiate to nerve cells.^36^ On the contrary, a high incidence of PTEN loss‐of‐function by mutation or deletion is common in patients with T‐cell acute lymphoblastic leukaemia, which is characterized by abnormal high proliferation and low differentiation.^37^ It is well understood that PTEN is a negative regulator of phosphoinositide 3‐kinase (PI3K) via interacting with phosphatidylinositol‐3,4,5‐triphosphate (PIP3) and thereby inhibiting the activation of Akt.^34^ It is generally accepted that activated Akt in turn leads to the phosphorylation of mTOR, which negatively regulates autophagy by phosphorylating uncoordinated‐51‐like kinases 1 (ULK1).^38^ Therefore, it is essential to investigate the effects of miR‐19b‐3p overexpression on PTEN and its downstream signalling in BMSC‐derived osteoblasts. In accordance with the data from the femurs of SCI rats, PTEN expression was decreased with miR‐19b‐3p up‐regulation in BMSC‐derived osteoblasts. Moreover, miR‐19b‐3p overexpression accelerated Akt and mTOR phosphorylation, restrained autophagy activation and thereby diminishing the osteogenic differentiation ability of BMSCs. The key role of autophagy activation in osteogenic differentiation has been well expounded. For example, inhibiting autophagy activity by 3‐methyladenine (3‐MA) decreases BMSC osteogenic levels,^39^ inversely, autophagy inducer rapamycin has the opposite effect.^40^ We also observed that the administration of rapamycin, an autophagy inducer, could abrogate the osteogenic inhibition of miR‐19b‐3p over‐expression. Furthermore, emerging evidences have demonstrated that the proliferation ability of BMSCs is enhanced with autophagy activation, which also contributes to better osteogenesis.^41^ However, due to the limitation of experimental methods, it is difficult to specifically detect the relationship between stem cell activity and autophagy in vivo. More efforts should be put into practice to monitor this relationship in the future. The above‐mentioned results indicated that miR‐19b‐3p inhibited osteogenic differentiation of BMSCs through down‐regulating autophagy via PTEN/Akt/mTOR signalling pathway. Besides, the therapeutic effects of miR‐19b‐3p inhibition on bone loss after SCI were verified in SCI rats with antagomir‐19b‐3p treatment. Thus, our results proved that the deteriorative effects of miR‐19b‐3p on SCI‐related bone loss were strongly related to autophagy inhibition via PTEN/Akt/mTOR signalling pathway.

In conclusion, our study indicated that miR‐19b‐3p overexpression was one of the initiation factors of bone loss after SCI, which was attributed to its inhibiting effects on osteogenic differentiation of BMSCs through autophagy via PTEN/Akt/mTOR signalling pathway. Despite additional explorations are still essential, our present data suggest that miR‐19b‐3p is one of the possible therapeutic targets for bone loss after SCI.

## CONFLICT OF INTEREST

The authors declared no conflict of interest.

## AUTHOR CONTRIBUTION

**Da Liu:** Investigation (lead). **Bo Wang:** Methodology (lead). **Min Qiu:** Formal analysis (lead). **Ying Huang:** Conceptualization (lead).

## Data Availability

All experimental data can be obtained and requested from the corresponding author when necessary.
